# Gli1+ Osteogenic Progenitors Contribute to Condylar Development and Fracture Repair

**DOI:** 10.3389/fcell.2022.819689

**Published:** 2022-03-07

**Authors:** Shuo Chen, Lin Lan, Jie Lei, Yang He, Yi Zhang

**Affiliations:** ^1^ Department of Oral and Maxillofacial Surgery, Peking University School and Hospital of Stomatology, Beijing, China; ^2^ National Center of Stomatology, Beijing, China; ^3^ National Clinical Research Center for Oral Disease, Beijing, China; ^4^ National Engineering Laboratory for Digital and Material Technology of Stomatology, Beijing, China; ^5^ Center for TMD and Orofacial Pain, Peking University School, Hospital of Stomatology, Beijing, China

**Keywords:** Gli1+ cells, mandibular condyle, development, fracture healing, wnt signaling

## Abstract

The condyle plays a pivotal role in mandible development, which is regulated by various signaling molecules. The hedgehog (Hh) signaling pathway is known to modulate several processes during bone formation. However, the role of Gli1, as the read-out of Hh signaling activity, in condylar development and fracture healing has not been clarified. In this study, we discovered that a population of Gli1+ cells residing immediately below the cartilage functions as osteogenic progenitors by using *Gli1-Cre* ^*ERT2*^ *;tdTomato* mice. These Gli1+ cells contributed to nearly all osteoblasts in the subchondral bone during condyle postnatal development. Interestingly, Gli1-lineage cells could differentiate into osteoblasts and chondrocytes during fracture healing. Inhibiting Wnt/β-catenin signaling downregulated the proliferation and differentiation of Gli1+ cells *in vitro*. These findings suggest that Gli1+ progenitor cells participate in not only normal bone formation but also fracture healing; moreover, these cells may provide a potential target for promoting bone regeneration of the mandible.

## Introduction

The condyle is an important growth site of the mandible, which plays an essential role in mastication and speech (Ohrbach and Dworkin, 2016). Condylar cartilage promotes mandible growth through endochondral bone formation (Hinton et al., 2017). The chondrogenesis and subsequent endochondral ossification observed at the mandibular condyle are regulated by various signaling molecules (Owtad et al., 2013). Indian hedgehog (Ihh) signaling critically regulates osteoblast differentiation during condylar embryonic and postnatal (PN) development (Kurio et al., 2018; Bechtold et al., 2019). Global *Ihh* knockout at the embryonic stage leads to the complete absence of normal functional discs and lubricin-expressing joint cavities, as well as condylar cartilage dysplasia (Shibukawa et al., 2007). Ablation of Ihh in the cartilage of juvenile/early adult mice compromises chondroprogenitor organization and function and results in reduced chondroprogenitor and chondrocyte proliferation (Kurio et al., 2018).

Gli1 functions as the read-out of endogenous hedgehog (Hh) signaling activity and a transcription factor that regulates the expression of Hh target genes (Sun et al., 2020). However, few studies have reported the physiological function of Gli1 in the maintenance or differentiation of osteoblast progenitors in PN mice. Recent studies have used genetic lineage tracing to demonstrate that Gli1 marks stem/progenitor cells in the long bones, craniofacial bones, incisors, and periodontal ligaments of adult mice (Zhao et al., 2014; Zhao et al., 2015; Shi et al., 2017; Men et al., 2020). However, the potential relationship between Gli1 and PN skeletal progenitors in the condyle has not been investigated.

In this study, we revealed that Gli1+ cells residing immediately below the cartilage are osteogenic mesenchymal progenitors by using *Gli1-Cre* ^*ERT2*^ *;tdTomato* mice. These Gli1+ cells contribute to nearly all of the osteoblasts in the subchondral bone during condyle PN development. In addition, Gli1 lineage cells differentiated into osteoblasts and chondrocytes during condylar fracture healing. Therefore, Gli1+ progenitor cells participate in normal bone formation and fracture healing.

## Materials and Methods

### Animals

*Gli1-Cre* ^*ERT2*^ knock-in (JAX#007913) (Ahn and Joyner, 2004), *ROSA26* ^*loxp-STOP-loxp-tdTomato*^ conditional reporter (JAX#007905) (Madisen et al., 2010), and *Gli1-LacZ* heterozygote (JAX#008211) (Bai et al., 2002) were used in this study. The mice were housed in pathogen-free conditions and analyzed under a mixed background. Male and female mice were used for analysis. All experiments were approved by the Institutional of Animal Care and Use Committee of Peking University.

### Tamoxifen Administration

Tamoxifen (T5648, Sigma, St. Louis, MO, United States) was dissolved in corn oil (C8267, Sigma) to a concentration of 20 mg/ml and injected intraperitoneally at a dose of 1.5 mg/10 g body weight. Neonatal mice were injected once, and adult mice was injected for three consecutive days.

### Histological Analysis

Mouse mandibles were dissected, fixed overnight in 4% paraformaldehyde, and then decalcified with 10% EDTA (pH 7.4) for 2–4 weeks. The samples were processed for paraffin embedding and then cut into sections with 6 μm thickness. Hematoxylin and eosin (H&E) staining was performed according to the standard protocol (Schmitz et al., 2010).

### Safranin O and Fast Green Staining

Paraffin embedded sections were subjected to Safranin O and fast green staining (G1371, Solarbio, Beijing, China) following standard protocols (Schmitz et al., 2010).

### Immunofluorescence Staining

The decalcified samples were dehydrated in 15% sucrose/PBS solution for 2 h, 30% sucrose/PBS for 2 h, and 30% sucrose/OCT (4,583, Sakura, Torrance, CA, United States) overnight at 4°C and then embedded in OCT. Cryosections measuring 8 μm in thickness were immunofluorescence-stained following standard protocols. The primary antibodies included Runx2 (1:100, #12556, Cell Signaling Technology, Danvers, MA, United States), Osterix (1:100, ab209484, Abcam, Cambridge, United Kingdom), β-galactosidase (β-gal; 1:200, ab9361, Abcam), osteocalcin (Ocn; 1:100, ab93876, Abcam), and Sox9 (1:100, ab185230, Abcam). Alexa Fluor 488 and Alexa Fluor 568 (1:200, Invitrogen, Waltham, MA, United States) were used as secondary antibodies. DAPI (62248, Invitrogen) was used for counterstaining. ImageJ was used to analyzed the ratio of Osterix+/tdTomato + cells to Osterix + cells.

Cell samples were plated in a 4-well chamber slide (PEZGS0416, Millipore). The slides were washed with PBS, immediately fixed with 4% paraformaldehyde for 15 min, and blocked with goat serum for 30 min at room temperature. The cells were incubated with the primary antibody (Ki67, 1:200, ab15580) at 4°C overnight, then incubated with secondary antibodies at room temperature for 1 h, stained with phalloidin for 20 min (1:50, A22287, ThermoFisher Scientific), and counterstained with DAPI.

### Cell Culture and Sorting

Condylar subchondral bones were obtained from 1-week-old *Gli1-Cre* ^*ERT2*^ *;tdTomato* mice 1 week after induction. The cartilage was meticulously dissected and removed with fine forceps. The condyle tissue was cut into tiny pieces and transferred into six-well culture plates with growth medium [α-MEM (12571-048, Thermo Fisher Scientific, Waltham, MA, United States) supplemented with 10% fetal bovine serum, 100 U/mL penicillin, and 100 U/mL streptomycin) in a 5% CO_2_ atmosphere at 37°C. After 7 days, the cells were digested with TrypLE (1897328, GIBCO) and then filtered through a 40 mm cell strainer (352,340, Falcon, NY, United States) to remove the remaining cell mass. tdTomato + cells were sorted via flow cytometry by using a FACS Aria Sorp cell sorter (BD Biosciences, NJ, United States). P2-cultured cells were used for colony formation and osteogenic differentiation assays.

### Colony Formation Assay

P2 tdTomato + cells were seeded at a density of 2000 cells per well into 24-well culture plates and left undisturbed for the first 2 days. Then, the growth medium was changed every other day with or without Wnt inhibitor (XAV939) supplementation (1.0 µM, S1180, Selleck, Houston, TX, United States). XAV939 was dissolved in dimethyl sulfoxide (DMSO) according to manufacturer’s recommendations and used in *in vitro* experiments, and the control group was treated with the same DMSO concentration. After 7 days, the culture plates were stained with a mixture of 0.1% toluidine blue and 2% paraformaldehyde. Colonies with >1 mm diameter were counted as a single colony cluster.

### Osteogenic Differentiation Assay

A total of 2 × 10^5^ tdTomato + cells were cultured in a 24-well plate and induced in osteogenic differentiation medium supplemented with 10 nM dexamethasone, 100 µM L-ascorbic acid phosphate, and 5 mM β-glycerophosphate (Sigma-Aldrich). XAV939 was added to the culture media of different groups. After 21 days of induction, mineralized nodules were detected by staining with 2% alizarin red S (400480250, ACROS Organics, Fair Lawn, NJ, United States). Alizarin red S crystals were dissolved in distilled water with 10% cetylpyridinium chloride and measured at 590 nm on a BioTek ELx808 system (BioTek Instruments, Vermont, United States).

### Western Blot

Cell were lysed using RIPA buffer supplemented with protease inhibitor for 30 min on ice. Protein extracts were loaded onto 10% (w/v) sodium dodecyl sulfate–polyacrylamide gels and then transferred to PVDF membranes. The membranes were blocked with 5% milk for 1 h and incubated with primary antibodies, including Runx2 (1:1,000, #12556, Cell Signaling Technology) and Ocn (1:1,000, ab93876, Abcam), at 4°C overnight. After the cells were incubated with HRP-conjugated secondary antibody for 1 h at room temperature, signals were detected through SuperSignal West Femto Maximum Sensitivity Substrate (34095, ThermoFisher Scientific), and images were acquired using Fusion Fx (Vilber Lourmat, France). Integrated density was measured by ImageJ for quantification analysis.

### Condyle Fracture and Sham Surgery

The surgical approach followed a previously described protocol (Chen et al., 2019). In brief, the mice were anesthetized by intraperitoneal injection of 10 μL/g 4% chloral hydrate. A pre-auricular incision was made on the left side. The parotid tissues were lifted, and the masseter muscle was blunt-dissected. The condyle neck was exposed and clipped by scissors. For the sham surgery, the condylar neck was exposed but kept intact. The incision was closed by suturing in layers. The coronal section was harvested for histological analysis.

### Statistical Analysis

Statistical analysis was performed using GraphPad Prism 6, and independent two-tailed Student’s t-tests were used to evaluate significant differences. Statistical data are presented as mean ± SD. *p* < 0.05 was considered statistically significant.

## Results

### Postnatal Gli1+ Cells are Spatially Located at the Superficial Layers of the Cartilage and Chondro-Osseous Junction

**FIGURE 1:**
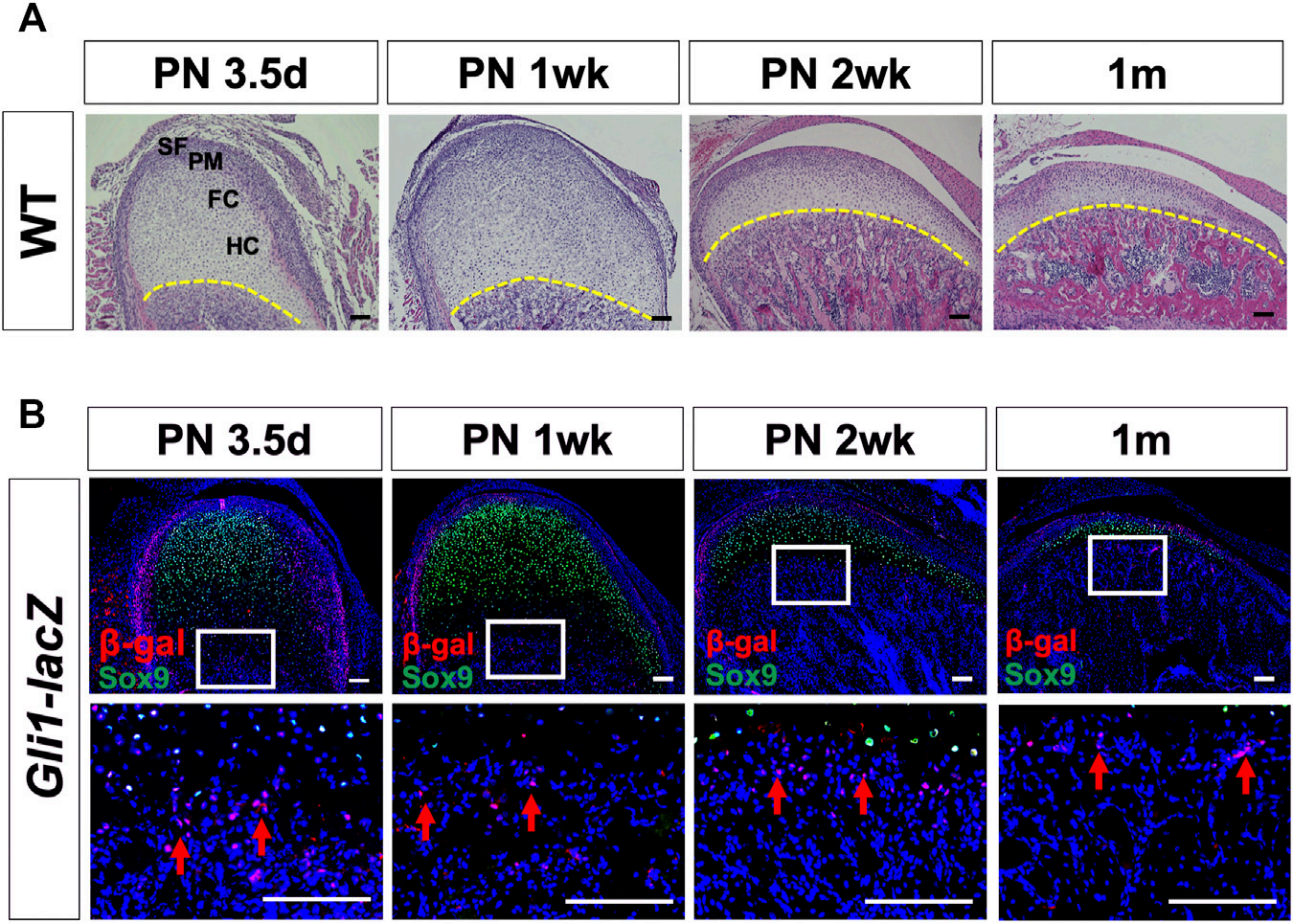
Postnatal Gli1+ cells are spatially located at the superficial layers of the cartilage and chondro-osseous junction. **(A)** Histological analysis of mandibular condyles from wild type (WT) mice at PN 3.5 days, 1 week, 2 weeks, and 1 month. **(B)** Sox9 and β-gal double-immunostaining of condyles from *Gli1-LacZ* mice at PN 3.5 days, 1 week, 2 weeks, and 1 month. The lower panel shows the high-magnification images of the white box insets in the upper panel. The yellow dotted line in **(A)** shows the chondro-osseous junction. Arrows in **(B)** show Gli1+ cells at the chondro-osseous junction. SF, superficial layer; PM, polymorphic zone; FC, flattened chondrocyte zone; HC, hypertrophic zone. *n* = 3 mice/group. Scale bars, 100 μm.

Gli1+ cells have been identified as a mesenchymal stem cell (MSC) population supporting craniofacial bone homeostasis and repair (Zhao et al., 2015; Guo et al., 2018). To investigate the expression of Gli1 in the mandibular condyle, we used wild type mice and collected samples at PN 3.5 days, 1 week, 2 weeks, and 1 month. H&E staining showed a superficial layer, a polymorphic progenitor cell layer, a flattened chondrocyte zone, and a hypertrophic chondrocyte zone at each stage (Figure 1A). The thickness of the condylar cartilage decreased from PN 3.5 days to 1 month old. Sox9, a master gene for chondrogenesis, is expressed in chondroprogenitor cells and chondrocytes (Liu et al., 2018; Sahu et al., 2020). We colocalized Sox9 by β-galactosidase (β-gal) immunostaining to characterize the expression pattern of Gli1 (Figure 1B). Few Gli1+ cells overlapped with Sox9+ chondroprogenitor cells and chondrocytes. Instead, Gli1+ cells consistently located at the superficial layer and chondro-osseous junction (Figure 1B). The superficial zone provides a niche for fibrocartilage stem cells that give rise to chondrocytes and osteocytes (Embree et al., 2016), and the chondro-osseous junction is the zone in which chondrogenesis ends and osteogenesis begins (Shen and Darendeliler, 2005; Jing et al., 2015). Therefore, the specific expression of Gli1+ in these two regions suggests that these cells may play a vital role in endochondral ossification.

### Gli1+ Cells at the Chondro-Osseous Junction Are Osteogenic Progenitors

**FIGURE 2:**
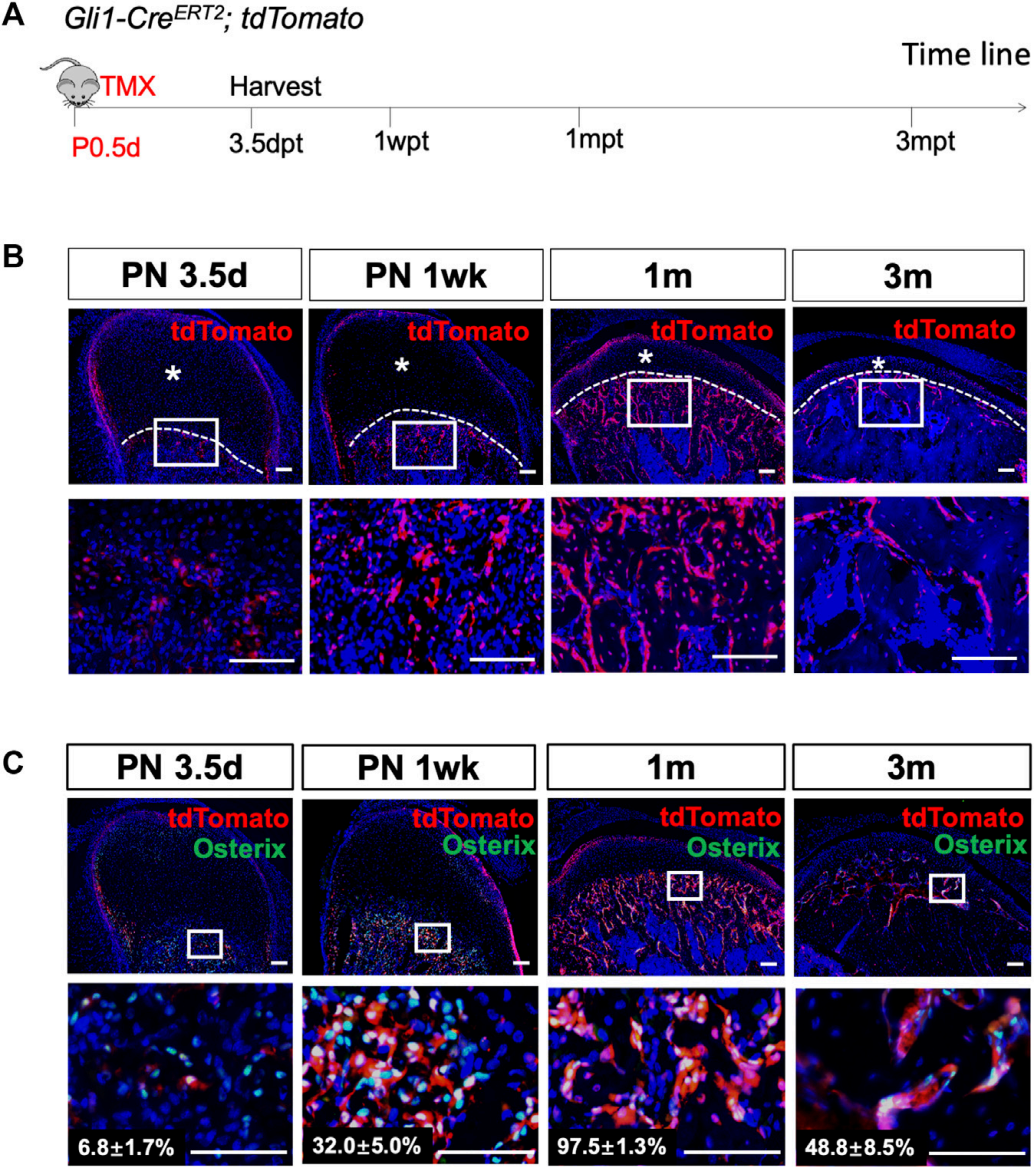
Gli1+ progenitor cells contribute to the osteoblast lineage during condylar postnatal development. **(A)** *Gli1-Cre* ^*ERT2*^ *;tdTomato* mice were induced by tamoxifen at PN 0.5 d, and the sample were harvested 3 days post-tamoxifen (3.5dpt), 1 week post-tamoxifen (1wpt), 1 month post-tamoxifen (1mpt), and 3 months post-induction (3mpt). **(B)** tdTomato immunostaining of condyles from *Gli1-Cre* ^*ERT2*^ *;tdTomato* mice 3 days, 1 week, 1 month and 3 months after tamoxifen induction at PN 0.5. **(C)** Osterix and tdTomato double-immunostaining of condyles from *Gli1-Cre* ^*ERT2*^ *;tdTomato* mice 3 days, 1 week, 1 month and 3 months after tamoxifen induction at PN 0.5. The white dotted line indicates the demarcation between the cartilage and subchondral bone. The lower panel represent high-magnification images of the white box insets in upper panel. Asterisks indicate the absence of tdTomato + signals. All data are presented as mean ± SD, *n* = 4 mice/group. Scale bars, 100 μm.

*Gli1-Cre* ^*ERT2*^ *;tdTomato* mice were generated, and lineage tracing was performed on neonatal mice to investigate the differentiation and migration of Gli1+ cells in bone formation during PN development (Figure 2A). Three days after induction, Gli1+ cells were found at the cartilage surface and chondro-osseous junction (Figure 2B, Supplementary Figure S1). Few Gli1+ cells were detected in the progenitor cell layer, chondrocyte zones, or subchondral bone, similar to the expression pattern of β-gal in PN3.5 *Gli1-LacZ* mice. Notably, 1 week after tracing, the Gli1+ progeny below the cartilage extended considerably toward the trabecular bone (Figure 2B). After 2 weeks of chasing, Gli1+ progeny expanded almost throughout the trabecular bone. The tdTomato + cells in the perichondrial layer also expanded but did not yet reach the chondro-osseous junction at this time (Supplementary Figure S1). One month after induction, descendants of the Gli1+ cells occupied nearly all of the trabecular bone, including not only osteoblasts on the bone surface but also osteocytes positioned in the bone matrix (Figure 2B). Interestingly, 3 months after induction, the number of Gli1+ progeny in the subchondral bone dramatically decreased (Figure 2B). Similarly, Gli1+ cells at the cartilage surface increased at 1 month and then decreased at 3 months of tracing (Figure 2B).

**FIGURE 3:**
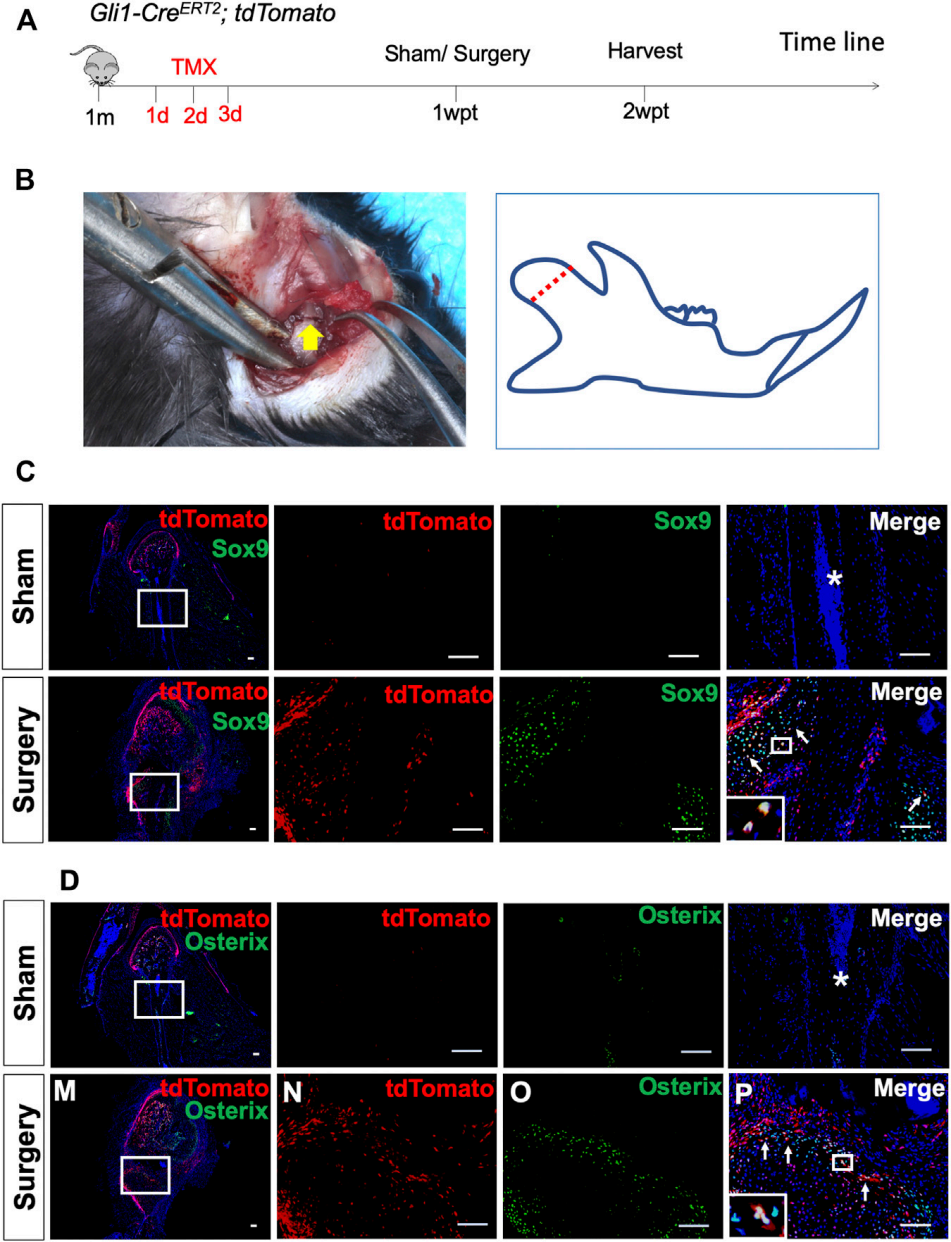
Gli1+ osteogenic progenitors contribute to condylar fracture repair. **(A)** *Gli1-Cre* ^*ERT2*^ *;tdTomato* mice were induced by tamoxifen at 1 month of age for three consecutive days. The surgery was performed 1 week post-tamoxifen (1wpt), and the samples were collected 2 weeks post-tamoxifen (2wpt). **(B)** The condyle neck was exposed and clipped by scissors. **(C)** Sox9 and tdTomato double-immunostaining of condyles from *Gli1-Cre* ^*ERT2*^ *;tdTomato* mice 1 week after sham surgery. **(D)** Osterix and tdTomato double-immunostaining of condyles from *Gli1-Cre* ^*ERT2*^ *;tdTomato* mice 1 week after sham surgery. White arrows indicate positive signals, and asterisks indicate the absence of signals in the sham/fracture sites. *n* = 3 mice/group. Scale bars, 100 μm.

Next, we focused on the Gli1+ cells located at the chondro-osseous junction. To verify whether these cells give rise to osteoblasts in the subchondral bone, we analyzed the colocalization of Gli1-linege cells with Osterix + osteoblasts. Three days after induction, less than 10% of the Osterix + cells beneath the articular cartilage overlapped with the Gli1+ cells (Figure 3C). One month after induction, almost 98% of the Osterix + cells appeared to be derived from the Gli1+ cells (Figure 3C). Approximately 49% of the Osterix + cells overlapped with Gli1+ progeny after 3 months of tracing (Figure 3C). Collectively, the Gli1+ cells at the chondro-osseous junction provide a major source of osteoblasts for trabecular bone formation in PN mice.

Condylar cartilage is categorized as secondary cartilage and undergoes adaptive changes in response to external stimuli even after natural growth (Delatte et al., 2004; Liu et al., 2012; Fujita et al., 2013; Chen et al., 2015). To investigate whether Gli1+ cells contribute to the osteoblast lineage during condylar remodeling further, we induced 4-week-old adult *Gli1-Cre* ^*ERT2*^ *;tdTomato* mice (Supplementary Figure S2A). One day after induction, several Gli1+ cells were scattered beneath the articular cartilage and overlapped with Runx2+ osteoblast lineage cells (Supplementary Figure S2B). One month after induction, Gli1+ cells expanded abundantly into the subchondral bone and gave rise to nearly all of the Runx2+ cells detected (Supplementary Figure S2B). Colocalization of the Gli1+ progeny and Runx2+ cells (Supplementary Figure S2B) could still be observed even after 9 months of tracing. These results suggest that Gli1+ cells residing at the chondro-osseous junction are osteogenic progenitors and could give rise to an osteoblast lineage during condylar PN development and adaptive remodeling.

### Gli1+ Osteogenic Progenitors Contribute to Fracture Repair

A condylar fracture was created to observe the healing process and test whether Gli1+ cells contribute to bone regeneration (Supplementary Figure S3A). The cartilage callus was initiated around the fractured site 1 week after surgery (Supplementary Figure S3B). Then, spongy bone formed to replace the cartilage tissue at 2 weeks (Supplementary Figure S3C). Finally, remodeling of the hard callus into a lamellar bone occurred to complete the healing process (Supplementary Figure S3D), which was comparable to sham surgery (Supplementary Figure S3E). We induced *Gli1-Cre* ^*ERT2*^ *;tdTomato* mice at 1 month of age, fractured the mandibular condyle 1 week after induction, and then harvested the sample 1 week after surgery (Figures 3A,B). No tdTomato fluorescence signal was detected at the sham-operated side (Figures 3C,D). At the fracture side, however, Gli1+ cells were activated, and their progeny migrated toward the fracture callus. Immunofluorescent staining confirmed that the Gli1+ progeny contributed to Sox9+ chondrocytes (Figure 3C), Osterix + osteoblasts (Figure 3D), and Ocn + osteoblasts (Supplementary Figure S4). These data confirm that Gli1 supports the skeletal progenitor pool for bone and cartilage formation during fracture healing.

### Gli1+ Cells Are Responsive to Wnt/β-Catenin Signaling

**FIGURE 4:**
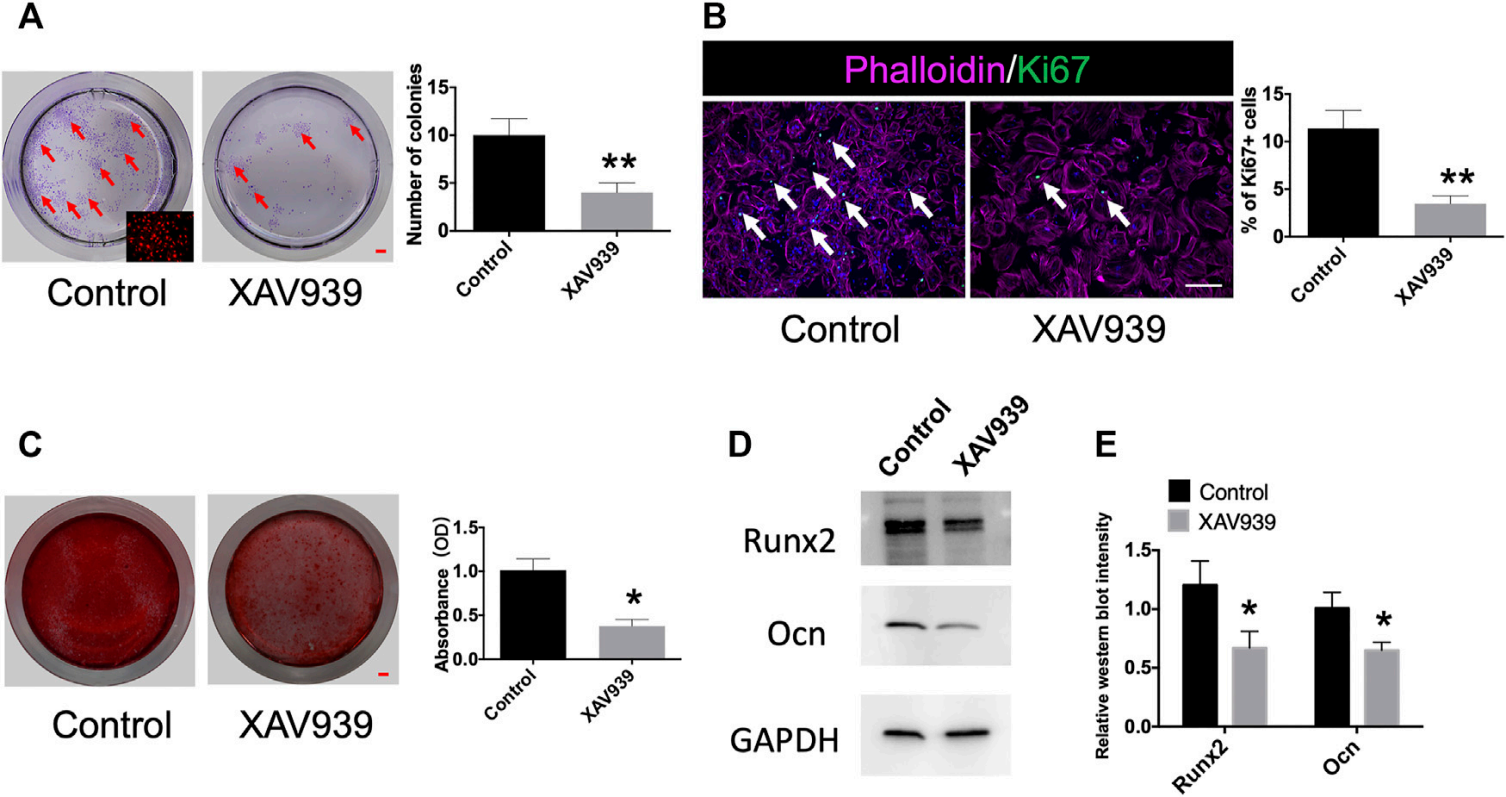
Gli1+ cells are responsive to Wnt/β-catenin signaling. **(A)** Colony formation and quantification analysis of Gli1+ cells from *Gli1-Cre* ^*ERT2*^ *;tdTomato* condyle. Red arrow indicates colony formation. **(B)** Ki67 and phalloidin double-immunostaining of Gli1+ cells and quantification analysis of Ki67 + cells from Gli1-CreERT2; tdTomato condyles. White arrow points to Ki67 + cells. **(C)** Alizarin red staining and absorbance analysis of Gli1+ cells from Gli1-CreERT2; tdTomato condyles. **(D)** Western blot of Runx2 and Ocn after osteogenic induction. **(E)** Quantitative analysis of Runx2 and Ocn protein levels. All experimental data were verified in three independent experiments. Scale bars **(A,C)** 1 mm, **(B)** 200 μm; *p < 0.05, **p < 0.01.

Recent studies have shown that Wnt/β-catenin signaling plays an important role in the osteogenic differentiation of MSCs (Li et al., 2019; Liang et al., 2021). To explore whether Gli1+ progenitors are regulated by Wnt/β-catenin signaling, we harvested tdTomato + cells from *Gli1-Cre* ^*ERT2*^ *;tdTomato* condyles 1 week after induction. The Gli1+ progeny was capable of colony formation and could differentiate into osteoblasts. The addition of Wnt inhibitor, XAV939, to the culture medium decreased colony formation considerably (Figure 4A). Immunofluorescence staining showed fewer Ki67 + cells after blocking Wnt signaling (Figure 4B). We assessed the extent of calcium deposition by alizarin red staining 3 weeks after osteogenic induction and found that Gli1+ cells deposited less calcium after addition of Wnt inhibitor compared with the control after osteogenic induction (Figure 4C). In addition, the control Gli1+ cells expressed significantly higher protein levels of Runx2 and Ocn than the Wnt inhibitor group (Figures 4D, E, Supplementary Figure S5). Taken together, the results indicate that Wnt/β-catenin signaling regulates the proliferation and differentiation of Gli1+ progenitor cells.

## Discussion

Condyle development and fracture repair represents an important but understudied topic in craniofacial research. Using the genetic lineage tracing technique, we discovered that Gli1+ cells residing at the chondro-osseous junction are a predominant source of osteoblasts during condyle PN development. The number of Gli1+ progeny decreased remarkably with age, thus suggesting that Gli1+ cells function as transient osteogenic progenitors. We also provided evidence that Gli1+ cells support the skeletal progenitor pool and contribute to not only chondrocytes but also osteoblasts during fracture healing. Our *in vitro* study indicated that Wnt/β-catenin signaling modulates the proliferation and differentiation of Gli1+ cells.

Multiple populations of MSCs have been identified in long bones and are marked by nestin and leptin receptors (Mendez-Ferrer et al., 2010; Zhou et al., 2014). MSCs surrounding bone marrow sinusoids are capable of multipotent differentiation and colony formation. Previous lineage tracing studies revealed that perisinusoidal MSCs give rise to osteoblasts, chondrocytes, and adipocytes. Another population of cells called skeletal stem cells (SSCs) was recently identified to be concentrated within the metaphysis of long bones (Chan et al., 2015; Worthley et al., 2015; Chan et al., 2018). SSCs specifically differentiate into osteoblasts, chondrocytes, and bone marrow stromal cells but not adipocytes. Shi et al. (2017) identified Gli1+ cells residing immediately beneath the growth plate as a population of osteogenic progenitors, which are essential for cancellous bone population. The expression pattern of Gli1+ cells uncovered in the condyle in the present work is similar but not identical to that of the metaphysis. In the long bone, Gli1+ cells are located at the articular cartilage surface, the upper layers of the growth plate, and the chondro-osseous junction. In the mandibular condyle, Gli1+ cells reside at the superficial layers of the cartilage and chondro-osseous junction. Both types of Gli1+ cells at the chondro-osseous junction were proved to be osteogenic progenitors. Notably, the number of Gli1+ progeny decreased in the subchondral bone 3 months after induction, which may be related to bone turnover. The mean lifespan of osteoblasts is approximately 12 days (Weinstein et al., 1998). The osteoblasts derived from Gli1+ cells that undergo apoptosis could account for the decreased Gli1+ progeny in the subchondral bone.

Gli1+ cells at the superficial layers of the condylar cartilage increased at 1 month and decreased at 3 months post-induction. This behavior is similar to that of Gli1+ cells in the upper layers of the growth plate but different from that of Gli1+ cells at the articular cartilage surface of the femur. Such variation is probably related to differences in the biological characteristics of condylar and epiphyseal cartilages. Condylar cartilage functions as an articular cartilage and a growth site, unlike femurs, which consist of a secondary ossification center at each apical end (Delatte et al., 2004). The longitudinal thickness of the condylar cartilage decreases with age (Kurio et al., 2018). Therefore, we speculated that the decrease in Gli1+ cells at the cartilage surface was related to the reduced cartilage thickness.

Gli1+ cells have been identified as MSCs/progenitor cells that are responsible for tissue/organ development, homeostasis, and injury repair in the craniofacial region. Gli1+ cells in the proximal region are typical MSCs in the mouse incisor (Zhao et al., 2014; Shi et al., 2019). These stem cells surround the neurovascular bundle and contribute to nearly all odontoblasts and dental pulp cells by supporting homeostasis and dentin regeneration (Chen et al., 2020). Zhao et al. revealed that Gli1+ cells within the suture mesenchyme as a main MSC population support craniofacial bone turnover and injury repair (Zhao et al., 2015). Yu et al. (2021) used a modified GelMA combined with Gli1+ MSCs and successfully regenerated functional cranial sutures that could correct skull deformities and rescue neurocognitive behavior deficits in *Twist1* ± craniosynostosis mice. In the present study, we uncovered a previously unknown population of Gli1+ cells at the chondro-osseous junction functioning as osteogenic progenitors in the condyle and contributing to bone formation during PN development and fracture repair. Increased tdTomato signals were also observed in the cartilage during fracture repair. The fibrous superficial zone has been identified as a niche harboring fibrocartilage stem cells (Embree et al., 2016). Therefore, we could not exclude the possibility that Gli1+ cells at the superficial layers may be activated and differentiated into osteoblasts and/or chondrocytes during fracture repair. Besides, we found that Gli1+ cells at the chondro-osseous junction gave rise to an osteoblast lineage during condylar remodeling. Condylar cartilage, also called secondary cartilage, could be distinguished from primary epiphyseal cartilage and is capable of adaptive remodeling in response to external stimuli. Therefore, these Gli1+ cells provide a potential target that could promote condylar growth even beyond natural growth.

Wnt signaling plays a critical role in bone formation and remodeling (Houschyar et al., 2018). Conditionally deleted β-catenin in Cxcl12 + bone marrow stromal cells results in remarkable reductions in bone volume and bone mineral density in the injured cortical bone compared with control mice (Matsushita et al., 2020). Blocking Wnt ligands from the osteoblastic lineage causes defects in bone development, while enhancing Wnt signaling by addition of Wnt-3a promotes fracture healing due to the increased proliferation and differentiation of skeletal stem/progenitor cells (Minear et al., 2010). We sorted the Gli1+ cells and cultured them *in vitro*. The cells exhibited self-renewal, colony-forming, and osteogenic differentiation capacities. However, proliferation and differentiation were significantly downregulated when Wnt signaling was inhibited. Thus, Wnt/β-catenin signaling may mediate the osteogenesis of Gli1+ cells.

In summary, we have uncovered a population of osteogenic progenitors that could be labeled Gli1 at the mandibular condyle. These Gli1+ cells reside at the chondro-osseous junction immediately beneath the cartilage. Gli1+ progenitors contribute to the osteoblast lineage during condylar PN development, homeostasis, and fracture repair. Wnt/β-catenin signaling may be a crucial driving force for the osteogenic differentiation of Gli1+ cells. Our study provides a potential target that could promote condylar growth and fracture healing.

## Data Availability

The original contributions presented in the study are included in the article/Supplementary Material, further inquiries can be directed to the corresponding authors.

## References

[B1] Ahn S. Joyner A. L. (2004). Dynamic Changes in the Response of Cells to Positive Hedgehog Signaling during Mouse Limb Patterning. Cell 118 (4), 505–516. 10.1016/j.cell.2004.07.023 15315762

[B2] Bai C. B. Auerbach W. Lee J. S. Stephen D. Joyner A. L. (2002). Gli2, but notGli1, Is Required for Initial Shh Signaling and Ectopic Activation of the Shh Pathway. Development 129 (20), 4753–4761. 10.1242/dev.129.20.4753 12361967

[B3] Bechtold T. E. Kurio N. Nah H.-D. Saunders C. Billings P. C. Koyama E. (2019). The Roles of Indian Hedgehog Signaling in TMJ Formation. Ijms 20 (24), 6300. 10.3390/ijms20246300 PMC6941023 31847127

[B4] Chan C. K. F. Gulati G. S. Sinha R. Tompkins J. V. Lopez M. Carter A. C. (2018). Identification of the Human Skeletal Stem Cell. Cell 175 (1), 43–56. 10.1016/j.cell.2018.07.029 30241615 PMC6400492

[B5] Chan C. K. F. Seo E. Y. Chen J. Y. Lo D. McArdle A. Sinha R. (2015). Identification and Specification of the Mouse Skeletal Stem Cell. Cell 160 (1-2), 285–298. 10.1016/j.cell.2014.12.002 25594184 PMC4297645

[B6] Chen S. Liu X. J. Li Z. L. Liang C. Wang X. X. Fu K. Y. (2015). Three-dimensional Evaluation of Condylar Morphology Remodeling after Orthognathic Surgery in Mandibular Retrognathism by Cone-Beam Computed Tomography. Beijing Da Xue Xue Bao Yi Xue Ban 47 (4), 703–707. 26284413

[B7] Chen S. He L.-h. Zhao L. Xiao E. He Y. Zhang Y. (2019). Effects of Articular Disc or Condylar Cartilage Resection on Mandibular Growth in Young Rats. Arch. Oral Biol. 97, 67–71. 10.1016/j.archoralbio.2018.10.005 30347348

[B8] Chen S. Jing J. Yuan Y. Feng J. Han X. Wen Q. (2020). Runx2+ Niche Cells Maintain Incisor Mesenchymal Tissue Homeostasis through IGF Signaling. Cel Rep. 32 (6), 108007. 10.1016/j.celrep.2020.108007 PMC7461627 32783935

[B9] Delatte M. Von den Hoff J. W. van Rheden R. E. M. Kuijpers-Jagtman A. M. (2004). Primary and Secondary Cartilages of the Neonatal Rat: the Femoral Head and the Mandibular Condyle. Eur. J. Oral Sci. 112 (2), 156–162. 10.1111/j.0909-8836.2004.00108.x 15056113

[B10] Embree M. C. Chen M. Pylawka S. Kong D. Iwaoka G. M. Kalajzic I. (2016). Exploiting Endogenous Fibrocartilage Stem Cells to Regenerate Cartilage and Repair Joint Injury. Nat. Commun. 7, 13073. 10.1038/ncomms13073 27721375 PMC5062541

[B11] Fujita T. Hayashi H. Shirakura M. Tsuka Y. Fujii E. Kawata T. (2013). Regeneration of Condyle with a Functional Appliance. J. Dent Res. 92 (4), 322–328. 10.1177/0022034513480795 23439718

[B12] Guo Y. Yuan Y. Wu L. Ho T.-V. Jing J. Sugii H. (2018). BMP-IHH-mediated Interplay between Mesenchymal Stem Cells and Osteoclasts Supports Calvarial Bone Homeostasis and Repair. Bone Res. 6, 30. 10.1038/s41413-018-0031-x 30345151 PMC6193039

[B13] Hinton R. J. Jing Y. Jing J. Feng J. Q. (2017). Roles of Chondrocytes in Endochondral Bone Formation and Fracture Repair. J. Dent Res. 96 (1), 23–30. 10.1177/0022034516668321 27664203 PMC5347428

[B14] Houschyar K. S. Tapking C. Borrelli M. R. Popp D. Duscher D. Maan Z. N. (2018). Wnt Pathway in Bone Repair and Regeneration - what Do We Know So Far. Front. Cel Dev. Biol. 6, 170. 10.3389/fcell.2018.00170 PMC6330281 30666305

[B15] Jing Y. Zhou X. Han X. Jing J. von der Mark K. Wang J. (2015). Chondrocytes Directly Transform into Bone Cells in Mandibular Condyle Growth. J. Dent Res. 94 (12), 1668–1675. 10.1177/0022034515598135 26341973 PMC4681473

[B16] Kurio N. Saunders C. Bechtold T. E. Salhab I. Nah H.-D. Sinha S. (2018). Roles of Ihh Signaling in Chondroprogenitor Function in Postnatal Condylar Cartilage. Matrix Biol. 67, 15–31. 10.1016/j.matbio.2018.02.011 29447948 PMC5910228

[B17] Li X. Liu D. Li J. Yang S. Xu J. Yokota H. (2019). Wnt3a Involved in the Mechanical Loading on Improvement of Bone Remodeling and Angiogenesis in a Postmenopausal Osteoporosis Mouse Model. FASEB j. 33 (8), 8913–8924. 10.1096/fj.201802711R 31017804 PMC9272758

[B18] Liang Y. Liu X. Zhou R. Song D. Jiang Y.-Z. Xue W. (2021). Chaetocin Promotes Osteogenic Differentiation via Modulating Wnt/Beta-Catenin Signaling in Mesenchymal Stem Cells. Stem Cell Int. 2021, 1–6. 10.1155/2021/8888416 PMC7886529 33628276

[B19] Liu C.-F. Angelozzi M. Haseeb A. Lefebvre V. (2018). SOX9 Is Dispensable for the Initiation of Epigenetic Remodeling and the Activation of Marker Genes at the Onset of Chondrogenesis. Development 145 (14). 10.1242/dev.164459 PMC6078338 30021842

[B20] Liu M.-Q. Chen H.-M. Yap A. U. J. Fu K.-Y. (2012). Condylar Remodeling Accompanying Splint Therapy: a Cone-Beam Computerized Tomography Study of Patients with Temporomandibular Joint Disk Displacement. Oral Surg. Oral Med. Oral Pathol. Oral Radiol. 114 (2), 259–265. 10.1016/j.oooo.2012.03.004 22769412

[B21] Madisen L. Zwingman T. A. Sunkin S. M. Oh S. W. Zariwala H. A. Gu H. (2010). A Robust and High-Throughput Cre Reporting and Characterization System for the Whole Mouse Brain. Nat. Neurosci. 13 (1), 133–140. 10.1038/nn.2467 20023653 PMC2840225

[B22] Matsushita Y. Nagata M. Kozloff K. M. Welch J. D. Mizuhashi K. Tokavanich N. (2020). A Wnt-Mediated Transformation of the Bone Marrow Stromal Cell Identity Orchestrates Skeletal Regeneration. Nat. Commun. 11 (1), 332. 10.1038/s41467-019-14029-w 31949165 PMC6965122

[B23] Men Y. Wang Y. Yi Y. Jing D. Luo W. Shen B. (2020). Gli1+ Periodontium Stem Cells Are Regulated by Osteocytes and Occlusal Force. Develop. Cel 54 (5), 639–654. 10.1016/j.devcel.2020.06.006 32652075

[B24] Méndez-Ferrer S. Michurina T. V. Ferraro F. Mazloom A. R. Macarthur B. D. Lira S. A. (2010). Mesenchymal and Haematopoietic Stem Cells Form a Unique Bone Marrow Niche. Nature 466 (7308), 829–834. 10.1038/nature09262 20703299 PMC3146551

[B25] Minear S. Leucht P. Jiang J. Liu B. Zeng A. Fuerer C. (2010). Wnt Proteins Promote Bone Regeneration. Sci. Transl. Med. 2 (29), 29ra30. 10.1126/scitranslmed.3000231 20427820

[B26] Ohrbach R. Dworkin S. F. (2016). The Evolution of TMD Diagnosis. J. Dent Res. 95 (10), 1093–1101. 10.1177/0022034516653922 27313164 PMC5004241

[B27] Owtad P. Park J. H. Shen G. Potres Z. Darendeliler M. A. (2013). The Biology of TMJ Growth Modification. J. Dent Res. 92 (4), 315–321. 10.1177/0022034513476302 23358678

[B28] Sahu N. Budhiraja G. Subramanian A. (2020). Preconditioning of Mesenchymal Stromal Cells with Low-Intensity Ultrasound: Influence on Chondrogenesis and Directed SOX9 Signaling Pathways. Stem Cel Res Ther 11 (1), 6. 10.1186/s13287-019-1532-2 PMC6942392 31900222

[B29] Schmitz N. Laverty S. Kraus V. B. Aigner T. (2010). Basic Methods in Histopathology of Joint Tissues. Osteoarthritis and Cartilage 18 (Suppl. 3), S113–S116. 10.1016/j.joca.2010.05.026 20864017

[B30] Shen G. Darendeliler M. A. (2005). The Adaptive Remodeling of Condylar Cartilage- A Transition from Chondrogenesis to Osteogenesis. J. Dent Res. 84 (8), 691–699. 10.1177/154405910508400802 16040724

[B31] Shi C. Yuan Y. Guo Y. Jing J. Ho T. V. Han X. (2019). BMP Signaling in Regulating Mesenchymal Stem Cells in Incisor Homeostasis. J. Dent Res. 98 (8), 904–911. 10.1177/0022034519850812 31136721 PMC6616121

[B32] Shi Y. He G. Lee W.-C. McKenzie J. A. Silva M. J. Long F. (2017). Gli1 Identifies Osteogenic Progenitors for Bone Formation and Fracture Repair. Nat. Commun. 8 (1), 2043. 10.1038/s41467-017-02171-2 29230039 PMC5725597

[B33] Shibukawa Y. Young B. Wu C. Yamada S. Long F. Pacifici M. (2007). Temporomandibular Joint Formation and Condyle Growth Require Indian Hedgehog Signaling. Dev. Dyn. 236 (2), 426–434. 10.1002/dvdy.21036 17191253

[B34] Sun M. R. Chung H. M. Matsuk V. Fink D. M. Stebbins M. J. Palecek S. P. (2020). Sonic Hedgehog Signaling in Cranial Neural Crest Cells Regulates Microvascular Morphogenesis in Facial Development. Front. Cel Dev. Biol. 8, 590539. 10.3389/fcell.2020.590539 PMC7575766 33117819

[B35] Weinstein R. S. Jilka R. L. Parfitt A. M. Manolagas S. C. (1998). Inhibition of Osteoblastogenesis and Promotion of Apoptosis of Osteoblasts and Osteocytes by Glucocorticoids. Potential Mechanisms of Their Deleterious Effects on Bone. J. Clin. Invest. 102 (2), 274–282. 10.1172/JCI2799 9664068 PMC508885

[B36] Worthley D. L. Churchill M. Compton J. T. Tailor Y. Rao M. Si Y. (2015). Gremlin 1 Identifies a Skeletal Stem Cell with Bone, Cartilage, and Reticular Stromal Potential. Cell 160 (1-2), 269–284. 10.1016/j.cell.2014.11.042 25594183 PMC4436082

[B37] Yu M. Ma L. Yuan Y. Ye X. Montagne A. He J. (2021). Cranial Suture Regeneration Mitigates Skull and Neurocognitive Defects in Craniosynostosis. Cell 184 (1), 243–256. 10.1016/j.cell.2020.11.037 33417861 PMC7891303

[B38] Zhao H. Feng J. Ho T.-V. Grimes W. Urata M. Chai Y. (2015). The Suture Provides a Niche for Mesenchymal Stem Cells of Craniofacial Bones. Nat. Cel Biol 17 (4), 386–396. 10.1038/ncb3139 PMC4380556 25799059

[B39] Zhao H. Feng J. Seidel K. Shi S. Klein O. Sharpe P. (2014). Secretion of Shh by a Neurovascular Bundle Niche Supports Mesenchymal Stem Cell Homeostasis in the Adult Mouse Incisor. Cell Stem Cell 14 (2), 160–173. 10.1016/j.stem.2013.12.013 24506883 PMC3951379

[B40] Zhou B. O. Yue R. Murphy M. M. Peyer J. G. Morrison S. J. (2014). Leptin-receptor-expressing Mesenchymal Stromal Cells Represent the Main Source of Bone Formed by Adult Bone Marrow. Cell Stem Cell 15 (2), 154–168. 10.1016/j.stem.2014.06.008 24953181 PMC4127103

