# Supplementary material for: Gli1+ Osteogenic Progenitors Contribute to Condylar Development and Fracture Repair
Source: Front Cell Dev Biol. 2022 Mar 7;10:819689. doi: 10.3389/fcell.2022.819689 (PMC8940214; doi:10.3389/fcell.2022.819689)
Supplement: Supplementary file 1 [file DataSheet1.pdf]

Supplementary Material

1. Supplementary Figures

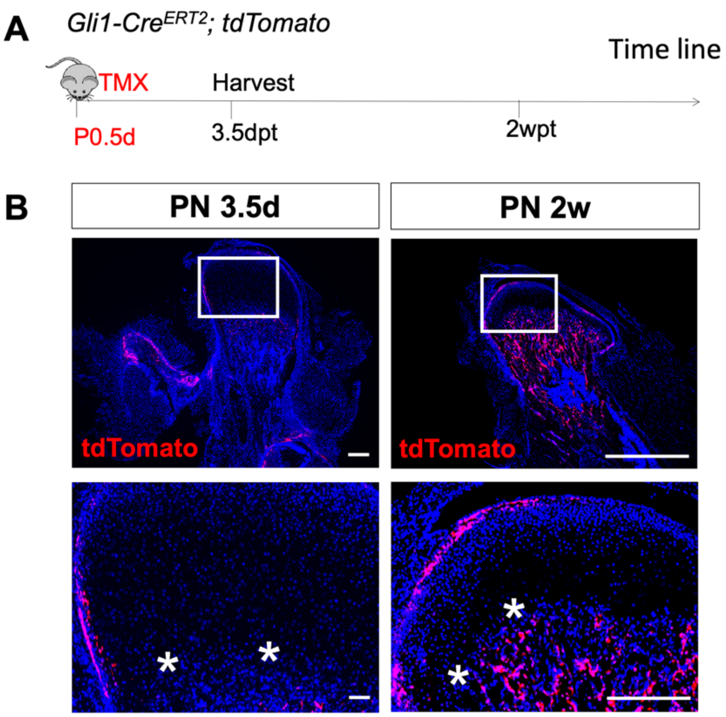

Figure S1. The migration of Gli1+ progeny cells during condylar postnatal development.

(A) *Gli1-Cre*^{ERT2}*;tdTomato* mice were induced by tamoxifen at PN 0.5d, and the sample were harvested 3 days post- tamoxifen (3.5dpt) and 2 week post- tamoxifen (2wpt).

(B) tdTomato immunostaining of condyles from *Gli1-Cre*^{ERT2}*;tdTomato* mice 3 days and 2 weeks after tamoxifen induction at PN 0.5d.

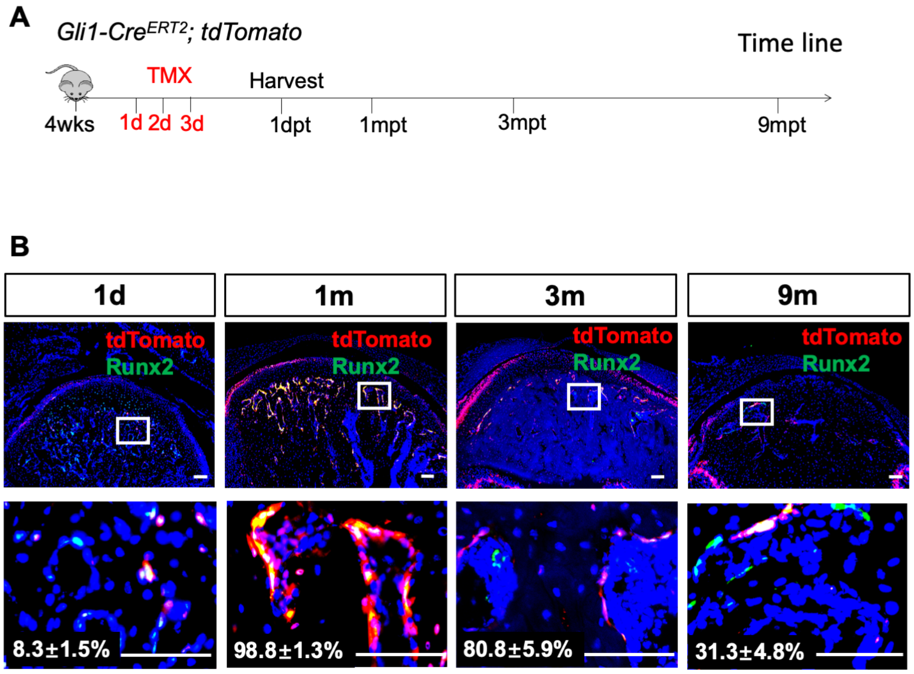

Figure S2. Gli1+ progenitor cells contribute to the osteoblast lineage during condylar adaptive remodeling.

(A) *Gli1-Cre^{ERT2};tdTomato* mice were induced by tamoxifen at 1 month of age for 3 consecutive days. The samples were collected 1day post- tamoxifen (1dpt), 1month post- tamoxifen (1mpt), 3months post- tamoxifen (3mpt), and 9months post- tamoxifen (9mpt).

(B) Runx2 and tdTomato double-immunostaining of condyles from *Gli1-Cre^{ERT2};tdTomato* mice 1day post- tamoxifen (1dpt), 1month post- tamoxifen (1mpt), 3months post- tamoxifen (3mpt), and 9months post- tamoxifen (9mpt). (M) presents a high-magnification image of the inset in (I).

The lower panel represent high-magnification images of the insets in upper panel. All data are presented as mean ± SD, $n = 4$. Scale bars, 100 μm.

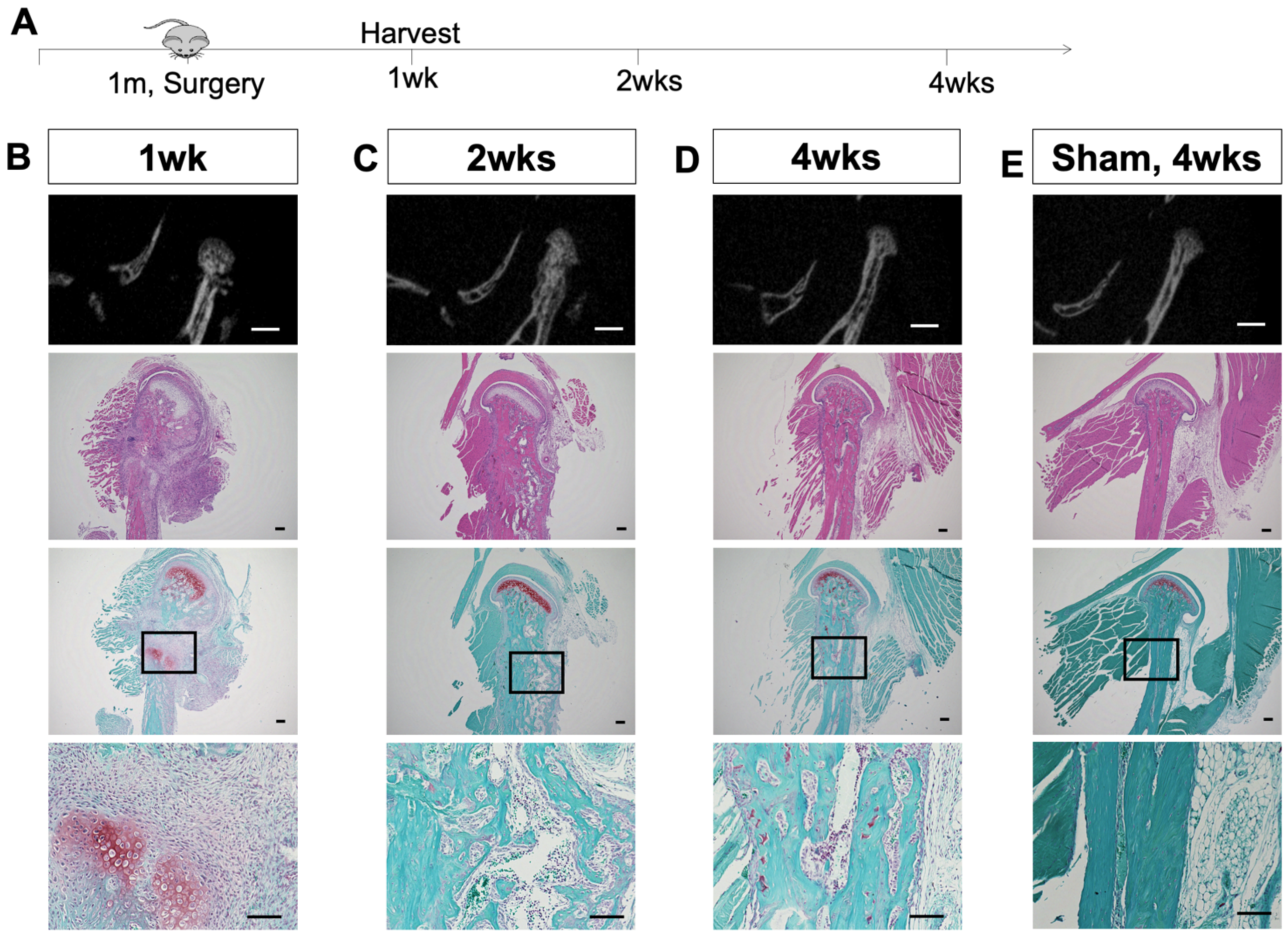

Figure S3. Healing process of condylar fracture.

(A) The surgery was performed at 1 month of age, and the samples were collected 1 week, 2 weeks and 4 weeks after surgery.

(B) Micro-CT imaging and H&E, Safranin O, and fast green staining results obtained 1 week after condylar fracture.

(C) Micro-CT imaging and H&E, Safranin O, and fast green staining results obtained 2 weeks after condylar fracture.

(D) Micro-CT imaging and H&E, Safranin O, and fast green staining results obtained 4 weeks after condylar fracture.

(E) Micro-CT imaging and H&E, Safranin O, and fast green staining results obtained 4 weeks after sham surgery.

Scale bars in Micro-CT are 600 μm; others, 100 μm.

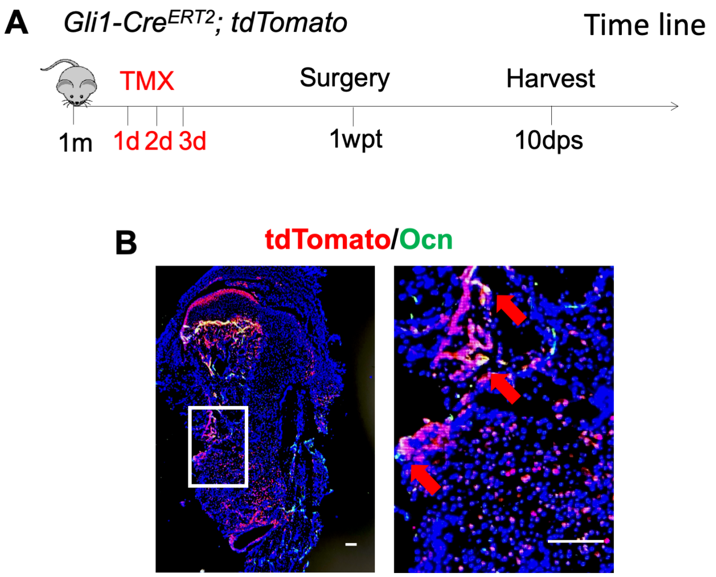

Figure S4. Gli1+ osteogenic progenitors contribute to Ocn+ osteoblasts during condylar fracture repair.

(A) *Gli1-Cre^{ERT2};tdTomato* mice were induced by tamoxifen at 1 month of age for 3 consecutive days. The surgery was performed 1week post- tamoxifen (1wpt), and the samples were collected 10 days post-surgery (10 dps).

(B) Ocn and tdTomato double-immunostaining of condyles from *Gli1-Cre^{ERT2};tdTomato* mice 10 days after surgery. Red arrows point to the overlap between Ocn and tdTomato signals.

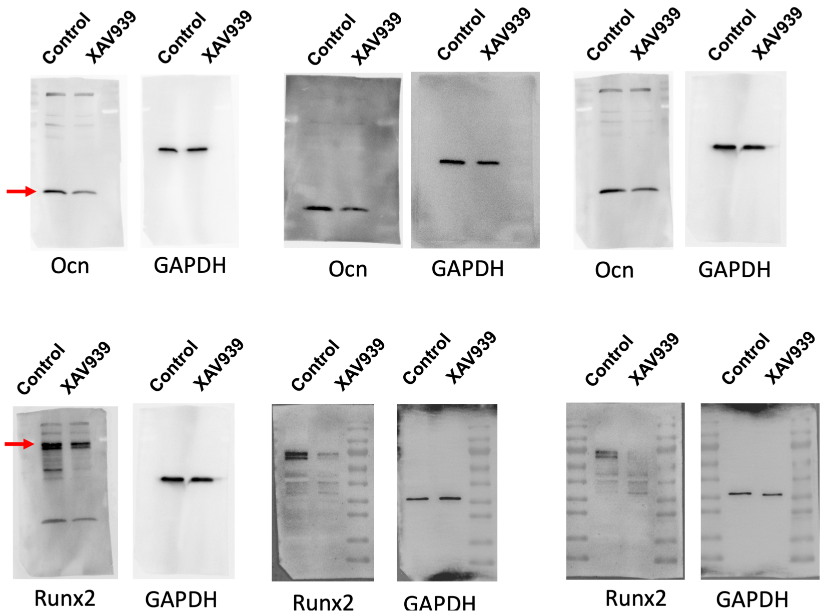

Figure S5. The uncropped images of Runx2 and Ocn Western blot analysis.

Red arrows point to the target proteins.
